# Compression of the Aorta in Uterine Hæmorrhage

**Published:** 1831-01-01

**Authors:** 


COMPRESSION OF THE AORTA IN UTERINE HÆMORRHAGE. By M. LATOUR.

*Case* 1. A female, aged 28 years, of delicate constitution, had miscarried in the sixth month of utero-gestation, without any inconvenience, and again became pregnant. In the course of this pregnancy she was seized with pneumonia, and when the inflammation was subdued, the motions of the fœtus ceased, but uterine contraction did not succeed till eight days afterwards. It was not till after thirty-six hours of lingering labour that the os uteri was so far dilated as to authorise the rupture of the membranes. It was found to be a cross presentation—the feet were seized—and a putrid fœtus of six months was taken away. Uterine hæmorrhage ensued—the placenta was extracted. The flooding continued, and the uterus became filled with blood. A deadly pallor ensued, and the patient said she was dying. She was quickly placed horizontal, and M. Latour made pressure on the aorta below the umbilicus, which was easily effected, as the patient was very thin. In a quarter of an hour the hæmorrhage ceased, and the womb was evidently contracting. M. Latour now thought that the compression of the aorta might be dispensed with, and took off the pressure. The patient was kept perfectly horizontal all that day, and next day several coagula of blood came away. The author asks was the arrest of the hæmorrhage owing to the compression of the aorta, or to other causes? He is not positive, but thinks it was attributable to this mechanical measure.

*Case* 2. M. Latour was called, in company with two other physicians, to the wife of a medical man. In the 5th month of her pregnancy she had been seized with cystitis, to which succeeded a most obstinate vomiting, which lasted two months almost incessantly. About the 7th month of utero-gestation the motions of the fœtus ceased, and with them the vomitings. In two days after this, labour pains occurred—the fœtus was expelled in three hours—the placenta followed—and although the discharge was little more than ordinary, the patient complained that she felt herself sinking. She begged her husband to raise her head, which he had no sooner done than the action of the heart ceased—to beat no more. This case is related by the author to put accoucheurs on their guard respecting position, when females have been reduced by hæmorrhage, or worn down by other diseases previous to parturition.

*Case* 3. This case, no more than the preceding one bears directly on the subject of the paper, but nevertheless it is deserving of notice.

A female, pregnant for the third time, came under our author's care for uterine hæmorrhage, which had continued for a month, at first trifling, but ultimately almost constant. This hæmorrhage resisted every means employed to check it, and at the close of the fifth month, premature delivery took place. The fate of the second case had made such an impression on M. Latour's mind that he was greatly alarmed for the safety of his present patient, who was much worn down by the long-continued drain from the uterine vessels. He therefore kept her in a horizontal position, and, as a precautionary measure, made pressure on the aorta. In this instance, however, a phenomenon ensued, which obliged him to relinquish the compression. In the course of a few minutes after the vessel was obstructed by the pressure of the fingers, her face became flushed—acute headache came on, with difficulty of breathing, &c. These symptoms immediately disappeared when the pressure was taken off—and returned when it was re-applied. The author believes that compression of the aorta is not only an important mean of stopping uterine hæmorrhage—but that it would be very useful in dangerous faintings after loss of blood, by distributing the circulation more towards the brain, and thus keeping up vital excitement in that organ. Certainly where the leanness of the individual permits pressure to be made on the great trunk of the aorta, there can be no possible objection to the measure, as it does not counteract or prevent the other and more usual means of relief being put into execution.—REVUE MEDICALE.

